# Hypertension Programmed by Perinatal High-Fat Diet: Effect of Maternal Gut Microbiota-Targeted Therapy

**DOI:** 10.3390/nu11122908

**Published:** 2019-12-02

**Authors:** Chien-Ning Hsu, Chih-Yao Hou, Julie Y.H. Chan, Chien-Te Lee, You-Lin Tain

**Affiliations:** 1 Department of Pharmacy, Kaohsiung Chang Gung Memorial Hospital, Kaohsiung 833, Taiwan; chien_ning_hsu@hotmail.com; 2 School of Pharmacy, Kaohsiung Medical University, Kaohsiung 807, Taiwan; 3 Department of Seafood Science, National Kaohsiung University of Science and Technology, Kaohsiung 811, Taiwan; chihyaohou@gmail.com; 4 Institute for Translational Research in Biomedicine, Kaohsiung Chang Gung Memorial Hospital and Chang Gung University College of Medicine, Kaohsiung 833, Taiwan; jchan@cgmh.org.tw; 5 Division of Nephrology, Kaohsiung Chang Gung Memorial Hospital, Kaohsiung 833, Taiwan; ctlee33@cgmh.org.tw; 6 Department of Pediatrics, Kaohsiung Chang Gung Memorial Hospital and Chang Gung University College of Medicine, Kaohsiung 833, Taiwan

**Keywords:** developmental programming, gut microbiota, hypertension, prebiotic, probiotic, renin-angiotensin system, short chain fatty acid, trimethylamine N-oxide

## Abstract

Hypertension can originate in early life caused by perinatal high-fat (HF) consumption. Gut microbiota and their metabolites short chain fatty acids (SCFAs), trimethylamine (TMA), and trimethylamine N-oxide (TMAO) are involved in the development of hypertension. Despite the beneficial effects of prebiotic/probiotic on human health, little is known whether maternal use of prebiotics/probiotics could protect offspring against the development of hypertension in adulthood. We investigated whether perinatal HF diet-induced programmed hypertension in adult offspring can be prevented by therapeutic uses of prebiotic inulin or probiotic *Lactobacillus casei* during gestation and lactation. Pregnant Sprague–Dawley rats received regular chow or HF diet (D12331, Research Diets), with 5% w/w long chain inulin (PRE), or 2 × 10^8^ CFU/day *Lactobacillus casei* via oral gavage (PRO) during pregnancy and lactation. Male offspring (*n* = 8/group) were assigned to four groups: control, HF, PRE, and PRO. Rats were sacrificed at 16 weeks of age. Maternal prebiotic or probiotic therapy prevents elevated blood pressure (BP) programmed by perinatal HF consumption. Both prebiotic and probiotic therapies decreased the *Firmicutes* to *Bacteroidetes* ratio and renal mRNA expression of *Ace*, but increased abundance of genus *Lactobacillus* and *Akkermansia*. Additionally, prebiotic treatment prevents HF-induced elevation of BP is associated with reduced fecal propionate and acetate levels, while probiotic therapy restored several *Lactobacillus* species. Maternal probiotic or prebiotic therapy caused a reduction in plasma TMAO level and TMAO-to-TMA ratio. The beneficial effects of prebiotic or probiotic therapy on elevated BP programmed by perinatal HF diet are relevant to alterations of microbial populations, modulation of microbial-derived metabolites, and mediation of the renin-angiotensin system. Our results cast a new light on the use of maternal prebiotic/probiotic therapy to prevent hypertension programmed by perinatal HF consumption. The possibility of applying gut microbiota-targeted therapies as a reprogramming strategy for hypertension warrants further clinical translation.

## 1. Introduction

Perinatal nutrition plays a key role in organogenesis and fetal development. Excessive or insufficient consumption of a specific nutrient during pregnancy and lactation has been linked to developmental programming of various adult diseases [1]. Developmental programming is defined as the process by which an environmental insult applied during critical periods of early life causes long-term effects on the structure or function of an organism [2]. This notion is currently recognized as “developmental origins of health and disease” (DOHaD) [3]. Increased consumption of saturated fat has been associated with obesity-related disorders [4]. In this regard, perinatal high-fat (HF) diet leads to a variety of metabolic syndrome-related phenotypes in adult offspring, including hypertension [5,6]. On the contrary, the DOHaD concept also affords preventive strategies to reverse programmed processes before clinical phenotype is becoming evident, by so-called reprogramming [7].

Because nutritional insults in gestation create very similar outcome in adult offspring [1], there might be some common mechanisms contributing to the pathogenesis of programmed hypertension. Emerging evidence indicates that inappropriate activation of the renin-angiotensin system (RAS) and changes in composition of the gut microbiota are involved in the pathogenesis of hypertension [8,9,10,11,12]. Blockers of the classical angiotensin-converting enzyme (ACE)/angiotensin (Ang) II/angiotensin type 1 receptor (AT1R) axis are well-established drugs for the treatment of hypertension [13]. As another important element of the RAS, ACE2 appears to adjust angiotensin II type 2 receptor (AT2R) and angiotensin (1–7) receptor Mas in a way that opposes the development of hypertension [13]. Both axes of the RAS have been examined on their roles in developmental programming of hypertension [14,15]. Several microbial markers have been linked to hypertension, such as an increased *Firmicutes* to *Bacteroidetes* (F/B) ratio and a decreased abundance of beneficial microbes [8,9]. Additionally, gut microbiota-derived metabolites short-chain fatty acids (SCFAs), especially acetate, butyrate, and propionate, and trimethylamine N-oxide (TMAO) are involved in the development of hypertension [16,17,18]. The gut microbiota link between the mother and offspring is continued at and after birth by microbes present during delivery as well as postnatal breast milk [19]. Perinatal HF diet was reported to alter gut microbiota in adult offspring [20]. These findings suggest that gut microbiota and its metabolites not only impact hypertension development but also serve as a link between mothers consuming a diet high in saturated fat and programmed hypertension in their adult offspring. 

The consumption of probiotics (i.e., beneficial microbes) and prebiotics (i.e., indigestible dietary fiber that fuels the beneficial microbes) have been reported to modulate gut microbiota and treat a variety of diseases [21]. Our previous report demonstrated that maternal microbiota-targeted therapy protected adult rat offspring against programmed hypertension induced by perinatal high-fructose consumption [22]. However, little is known whether restoration of gut microbiome by probiotics or prebiotics could serve as a reprogramming strategy to prevent hypertension programmed by perinatal HF intake. The overall goal of this work was to use a perinatal HF diet-induced programmed hypertension model, to dissect the contributions of prebiotic inulin and probiotic *Lactobacillus casei* on gut microbiota and their metabolites, RAS, and programmed hypertension in adult offspring. 

## 2. Materials and Methods 

### 2.1. Animal Model 

The investigation conformed to the Institutional Animal Care and Use Committee of Kaohsiung Chang Gung Memorial Hospital (permit #201721408) that complies with the Guide for the Care and Use of Laboratory Animals of the National Institutes of Health. Twelve virgin Sprague Dawley (SD) rats (12–16 weeks old) were purchased from BioLASCO Taiwan Co., Ltd. (Taipei, Taiwan). Rats were housed under 12-h light/12-h dark conditions with a relative humidity of 55% in an Association for Assessment and Accreditation of Laboratory Animal Care International (AAALAC)-approved animal facility in our hospital. Female rats were caged with males until mating was confirmed by observation of a vaginal plug. Pregnant SD rats were randomly divided into four groups and fed as follows (*n* = 3/group): (1) control group received regular chow (CON; Fwusow Taiwan Co., Ltd., Taichung, Taiwan; 52% carbohydrates, 23.5% protein, 4.5% fat, 10% ash, and 8% fiber) for a total of six weeks during pregnancy and lactation, (2) HF group received chow supplemented 58% high-fat diet (D12331, Research Diets, Inc., New Brunswick, NJ, USA; 25.5% carbohydrate, 58% fat (hydrogenated coconut oil), 16.4% protein, and 0% fiber) during pregnancy and lactation, (3) PRE group received 58% HF diet plus 5% w/w long chain inulin (#I2255, Sigma, St. Louis, MO, USA) during pregnancy and lactation, and (4) PRO group received 58% HF diet plus 2 × 108 CFU/day *Lactobacillus casei* (Antibiophilus, Laboratoires Lyocentre, France) via gavage of 1 mL of prepared spore suspension in water using a blunt-tipped gavage needle during pregnancy and lactation. Except the PRO group, other rats received 1 mL of water (vehicle) via gavage. The doses of prebiotics and probiotics have been demonstrated to be beneficial in rats [22,23]. After birth, litters were culled to eight pups to standardize the received quantity of milk and maternal pup care. As men are more prone to hypertension at a younger age [24], only male offspring were used in subsequent experiments. All male offspring were weaned at 3 weeks of age, and onto the regular chow ad libitum from weaning to 16 weeks of age. After weaning, two rats were co-housed in a cage.

BP-2000 tail-cuff system (BP-2000, Visitech Systems, Inc., Apex, NC, USA) was used in conscious rats. BP was measured at 3 weeks of age as baseline and at intervals of four weeks beginning from 4 16 weeks of age [22]. The rats were acclimated to restraint and tail-cuff inflation for 1 week prior to the measurement, to ensure accuracy and reproducibility. BP measurements were taken at 13:00–17:00 h each day. Rats were placed on the specimen platform, and their tails were passed through tail cuffs and secured in place with tape. Following a 10-min warm-up period, 10 preliminary cycles were performed to allow the rats to adjust to the inflating cuff. For each rat, five cycles were recorded at each time point. Three stable measures were taken and averaged. The rats were sacrificed at 16 weeks of age. Heparinized blood samples were collected, and the kidneys were harvested and stored at −80 °C freezer for further analysis. Fresh feces samples were collected at 3 and 16 weeks of age, frozen, and stored at −80 °C until use. Plasma creatinine levels were determined by HPLC as described previously [25].

### 2.2. Gas Chromatography-Flame Ionization Detector (GC-FID) 

Fecal concentrations of acetate, butyrate, and propionate were measured using gas chromatography-mass spectrometry (GCMS-QP2010; Shimadzu, Kyoto, Japan) with flame ionization detector (FID) [26]. Analytical standard grades of acetate, propionate (both from Sigma-Aldrich, St. Louis, MO, USA), and butyrate (from Chem Service, West Chester, PA, USA) were used as internal standards. The working solutions of acetate, butyrate, and propionate were prepared at the concentration of 10 mM and stored at −20 °C freezer. Dry air, nitrogen, and hydrogen were supplied to the FID at 300, 20 and 30 mL/min, respectively. A 2-µL aliquot of sample was injected into the column. The inlet temperature was set at 200 °C. The FID temperature was set at 240 °C. The total running time was 17.5 min.

### 2.3. Liquid Chromatography–Mass Spectrometry (LC–MS/MS) Analysis

TMAO is formed from TMA, which is generated by the metabolism of gut microbiota from dietary precursors (e.g., choline and L-carnitine) [16]. TMAO and TMA can be metabolized to dimethylamine (DMA). Thus, simultaneous measuring of TMA, TMAO, and DMA and their combined ratios may understand the whole picture of TMA–TMAO metabolic pathway in the pathogenesis of hypertension. We analyzed plasma DMA, TMA, and TMAO levels by LC–MS/MS analysis using an Agilent 6410 Series Triple Quadrupole mass spectrometer (Agilent Technologies, Wilmington, DE, USA) equipped with an electrospray ionization source [27]. The multiple-reaction-monitoring mode was set up using characteristic precursor-product ion transitions to detect m/z 46.1→30, m/z 60.1→44.1, and m/z 76.1→58.1, for DMA, TMA, and TMAO, respectively. Separation was performed in the Agilent Technologies 1200 HPLC system consisting of autosampler and a binary pump. Chromatographic separation was performed on a SeQuant ZIC-HILIC column (150 × 2.1 mm, 5 µm; Merck KGaA, Darmstadt, Germany) protected by an Ascentis C18 column (2 cm × 4 mm, 5 µm; Merck KGaA, Darmstadt, Germany). Diethyl amine was added to samples as an internal standard. The mobile phase containing methanol with 15mmol/L ammonium formate (phase A) and acetonitrile (phase B) was used at a ratio of 20:80 (phase A: phase B); with the flow rate set as 0.3–1 mL/min. 

### 2.4. Analysis of Gut-Microbiota Composition

Metagenomic DNA was extracted from frozen fecal samples using a fecal DNA isolation kit according to manufacturer’s instructions (EasyPrep Stool Genomic DNA Kit, Biotools Co., Ltd., New Taipei City, Taiwan). Simple centrifugation processing was carried out to removes impurity, proteins, and other organic compounds. As described previously [26], all polymerase chain-reaction amplicons were mixed together for sequencing using an Illumina Miseq platform (Illumina, CA, USA) at the Genomic and Proteomic Core Laboratory, Kaohsiung Chang Gung Memorial Hospital (Kaohsiung, Taiwan). Amplicons were prepared according to the 16S Metagenomics Sequencing Library Preparation protocol (Illumina, CA, USA), and sequenced the variable V3-V4 regions of the 16S rRNA gene with the Illumina MiSeq platform (Illumina, CA, USA) in paired-end mode with 600-cycle sequencing reagent. Next generation sequencing data were analyzed with the Microbial Genomics Module of CLC Genomics Workbench 9.5.4 (Qiagen, Stockach, Germany). Sequencing was assigned to operational taxonomic units with a pairwise identity threshold of 97%, and taxonomic classification was determining using the Greengenes database. A median of 156411 raw sequencing reads and 128580 effective tag sequences per sample was obtained, respectively. The average effective rate was 82%. Taxonomic relative abundance profiles at the phylum and genus levels were compared using the Kruskal-Wallis test with Dunn’s post hoc test, to identify significantly different bacterial taxa among different groups. Alpha diversity was measured by Shannon index. Beta diversity was assessed by weighted or unweighted UniFrac distances. We used linear discriminant analysis (LDA) effect size to agnostically identify microbial biomarkers. The LDA score represents an estimation of the magnitude of the difference between grouping categories. For stringency, microbial biomarkers in our study were retained if an LDA significant threshold > 2 was shown.

### 2.5. Western Blot

Western blot analysis was performed using the methods published previously [26]. Kidney cortex homogenate (200 μg) was loaded on a 10–15% polyacrylamide gel and separated by electrophoresis (200 V, 90 min). Following transfer to a nitrocellulose membrane (GE Healthcare Bio-Sciences Corp., Piscataway, NJ, USA), the membranes were incubated with Ponceau S red (PonS) stain solution (Sigma-Aldrich, St. Louis, MO, USA) for 10 min on the rocker to verify equal loading. We used the following antibodies: for ACE2, rabbit anti-rat ACE2 (1:1000, overnight incubation; Santa Cruz Biotechnology, Santa Cruz, California, USA); for AT1R, a rabbit anti-rat AT1R antibody (1:500, overnight incubation; AB15552, Millipore, Billerica, MA, USA); for AT2R, a rabbit anti-rat AT2R antibody (1:250, overnight incubation; sc-9040, Santa Cruz Biotechnology, Santa Cruz, CA, USA); and for angiotensin (1-7) receptor MAS, a rabbit anti-rat MAS antibody (1:1000, overnight incubation; Santa Cruz Biotechnology). Following five washes with 0.1% Tween-Tris-buffered saline (TBS-T), the membranes were incubated for 1 h with horseradish peroxidase-labeled secondary antibody diluted 1:1000 in TBS-T. Bands were visualized using SuperSignal West Pico reagent (Pierce; Rockford, IL, USA) and quantified by densitometry as integrated optical density (IOD). IOD was then normalized to total protein PonS staining. The protein abundance was represented as IOD/PonS.

### 2.6. Quantitative Real-Time Polymerase Chain Reaction (PCR) 

RNA was extracted from kidney cortex according to previously described methods [26]. RNA concentration and quality were checked by measuring optical density at 260 and 280 nm. The complementary DNA (cDNA) product was synthesized using a MMLV Reverse Transcriptase (Invitrogen). Two-step quantitative real-time PCR was conducted using the QuantiTect SYBR Green PCR Kit (Qiagen, Valencia, CA, USA) and the iCycler iQ Multi-color Real-Time PCR Detection System (Bio-Rad, Hercules, CA, USA). We analyzed three components of RAS, including renin (Ren), angiotensinogen (Agt), and *Ace*. The 18S rRNA gene (Rn18s) was used as a reference. Sequences of primers used in this study were listed in Table 1. All samples were run in duplicate. RNA expression levels were normalized to 18S rRNA levels and calculated according to the ΔΔCt method.

### 2.7. Statistical Analysis 

Data are reported as the mean ± standard error of mean (SEM), with statistical significance inferred where *p* < 0.05. Most parameters were analyzed with one-way ANOVA with a Tukey post hoc test for multiple comparisons. BP was analyzed by two-way repeated-measures ANOVA and Tukey’s post hoc test. Analyses were performed using the Statistical Package for the Social Sciences (SPSS) software (Chicago, IL, USA).

## 3. Results

### 3.1. Effect of HF, Probiotic, and Prebiotic on Morphological Features and Blood Pressures

**Figure 1:**
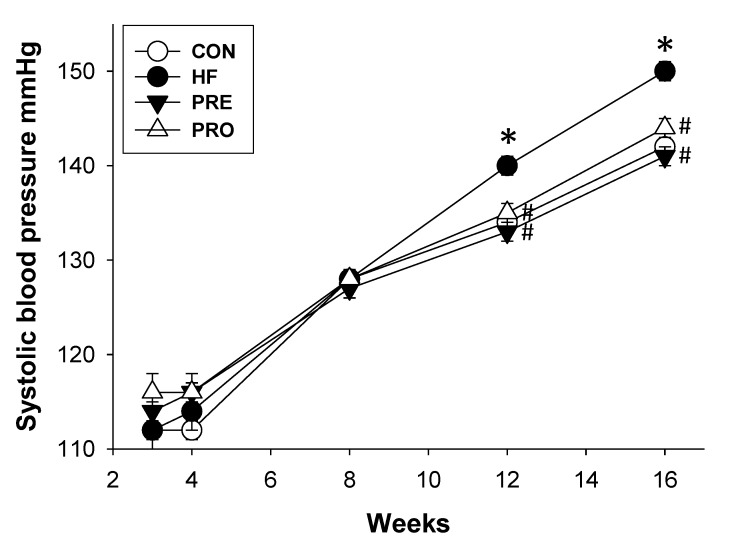
Effect of perinatal high-fat (HF) diet, prebiotic inulin (PRE), and probiotic *Lactobacillus casei* (PRO) on systolic blood pressure in male offspring from 3 to 16 weeks of age. * *p* < 0.05 versus CON; # *p* < 0.05 versus HF.

There was no dead pup in any group. As shown in Table 2, bodyweight, kidney weight, and kidney weight-to-BW ratio were not different among four groups. At 16 weeks of age, the systolic and diastolic BPs and mean arterial pressure of the HF group were higher than those in the control group (Table 2). A significant reduction in SBP was measured in the PRO and PRE groups versus the HF group. Figure 1 shows that HF exposure significantly increased the systolic BP from 12 to 16 weeks. A significant decrease of systolic BP was measured in the PRE and PRO groups versus the HF group from 12 to 16 weeks of age. Our data indicated that perinatal HF intake induced the elevation of BPs in offspring, which was prevented by prebiotic or probiotic therapy. HF diet caused a higher creatinine (Cr) level in HF group compared with control, whereas the Cr levels in the PRO and PRE group were comparable to controls (Table 2).

### 3.2. Effect of HF, Probiotic, and Prebiotic on Gut Microbiota-Derived Metabolites

Since SCFAs were found to be involved in the regulation of BP [12], we first investigated whether HF diet impaired SCFAs production while maternal probiotic or prebiotic therapy prevented it. At 3 weeks of age, we found that HF diet significantly decreased fecal concentrations of propionate in the HF, PRE, and PRO groups compared to controls (Table 3). Fecal acetate and butyrate levels were not different among four groups. Prebiotic therapy caused lower fecal concentrations of acetate than those in the controls at 16 weeks of age. Probiotic therapy resulted in a higher butyrate level in the PRO group compared to the PRE group. At 16 weeks of age, fecal propionate levels were not different among four groups.

Next, we determined plasma levels of TMAO, TMA, and DMA (Table 4). Maternal prebiotic or probiotic therapy significantly decreased plasma TMAO levels. Plasma TMA levels were higher in the HF, PRE, and PRO group compared to controls. Plasma DMA levels were lower in the HF and PRO groups than in the CON group. Based on the TMAO metabolic pathway, we created two conversion ratios, one being TMAO-to-TMA conversion ratio and the other being DMA-to-TMAO conversion ratio. Presumably, the TMAO-to-TMA ratio is proportional to the rate of TMAO synthesis, while the DMA-to-TMAO ratio is proportional to the rate of TMAO metabolism. A high TMAO-to-TMA ratio and a low DMA-to-TMAO ratio were expected to result in a cumulative increase of TMAO. Our results demonstrated that maternal probiotic or prebiotic therapy caused a lower TMAO-to-TMA ratio but a higher DMA-to-TMAO ratio than those in the HF group. These findings indicate that probiotic or prebiotic treatment prevent the accumulation of TMAO. 

### 3.3. Effect of HF, Probiotic, and Prebiotic on the RAS

**Figure 2:**
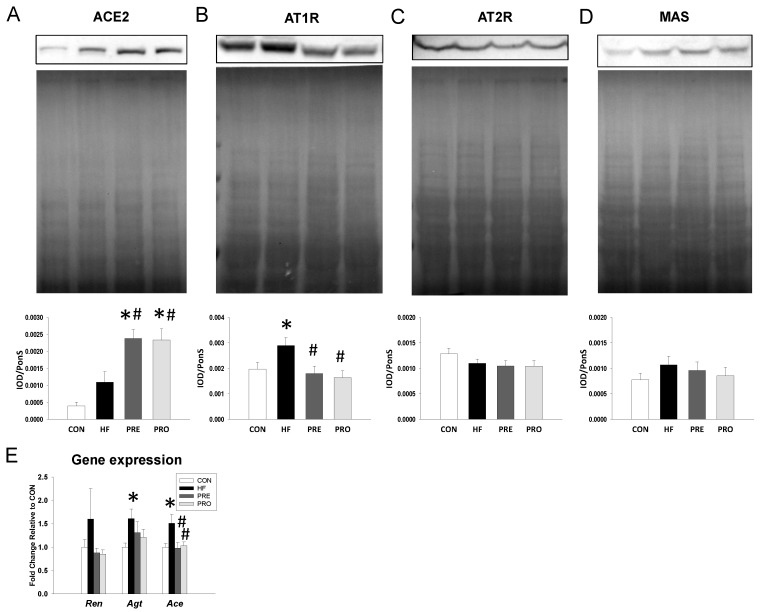
Effect of perinatal high-fat (HF) diet, prebiotic inulin (PRE), and probiotic *Lactobacillus casei* (PRO) on the RAS components in male offspring at 16 weeks of age. Representative western blots, Ponceau red staining, and relative abundance of (**A**) angiotensin-converting enzyme 2 (ACE2, 90 kDa), (**B**) angiotensin type 1 receptor (AT1R, 43 kDa), (**C**) angiotensin type 2 receptor (AT2R, 50 kDa), and (**D**) angiotensin (1-7) receptor MAS (37 kDa) bands in offspring kidneys at 16 weeks of age. (**E**) Gene expression of renin-angiotensin system components *Ren, Agt*, and *Ace* in male offspring at 16 weeks of age. *n* = 8/group. * *p* < 0.05 versus CON; # *p* < 0.05 versus HF.

We next evaluated the expression of RAS components in offspring kidneys (Figure 2). Renal protein levels of ACE2 were higher but AT1R were lower in the PRE and PROP group compared to those in the HF group (Figure 2B,C). However, AT2R and MAS protein abundance were not different among the four groups (Figure 2D,E). Additionally, HF diet significantly increased renal mRNA expression of Agt and *Ace* (Figure 2E). While renal *Ace* mRNA expression was lower in the PRO and PRE group than that in the HF group. 

### 3.4. Effect of HF, Probiotic, and Prebiotic on Gut Microbiota

**Figure 3:**
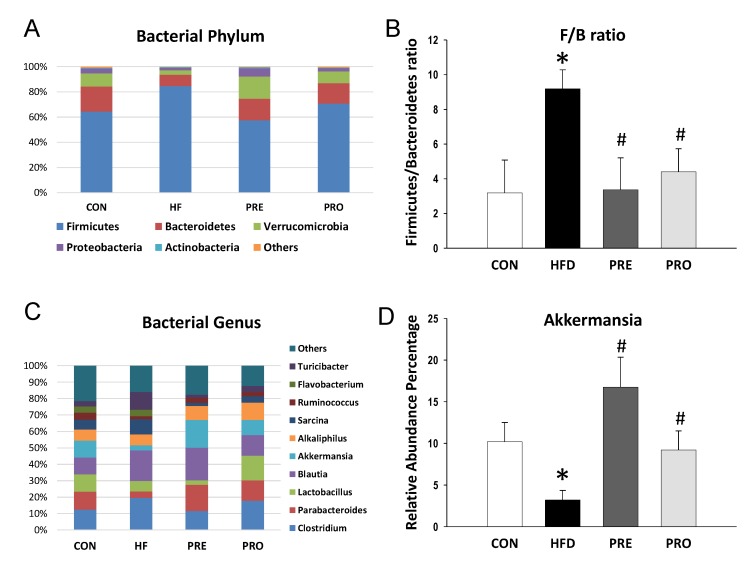
Effect of perinatal high-fat (HF) diet, prebiotic inulin (PRE), and probiotic *Lactobacillus casei* (PRO) on (**A**) relative abundances of the gut microbiota at the phylum level at 3 weeks of age; (**B**) the *Firmicutes* to *Bacteroidetes* ratio; (**C**) relative abundances of the gut microbiota at the genus level; and (**D**) the abundance of genus *Akkermansia*. * *p* < 0.05 versus CON; # *p* < 0.05 versus HF.

At 3 weeks of age, Shannon index analyses indicated that the alpha diversity did not differ significantly among four groups (All *p* > 0.05). Beta diversity was assessed by weighted or unweighted UniFrac distances, which did not reach significance between groups at 3 weeks of age. The main phyla detected included *Firmicutes, Bacteroidetes, Verrucomicrobia, Proteobacteria,* and *Actinobacteria* (Figure 3A). HF diet caused an increase in the phylum *Firmicutes* (85.6 ± 1.1% versus 64.3 ± 2.6%; *p* < 0.05) and a decrease in *Bacteroidetes* (9.2 ± 1% versus 20.2 ± 1.4%; *p* < 0.05), *Verrucomicrobia* (3.4 ± 1.2% versus 10.3 ± 2.3%; *p* < 0.05), and *Proteobacteria* (1.9 ± 0.1% versus 3.8 ± 0.3%; *p* < 0.05) in the HF group compared with controls. These HF diet-induced changes in phylum *Firmicutes* (57.5 ± 5.1%; *p* < 0.05 versus HF), *Bacteroidetes* (17.1 ± 2.8%; *p* < 0.05 versus HF), *Verrucomicrobia* (17.5 ± 3.8%; *p* < 0.05 versus HF), and *Proteobacteria* (6.7 ± 2.2%; *p* < 0.05 versus HF) were prevented by prebiotic therapy. A similar pattern of the results for prebiotic therapy was obtained in probiotic therapy. The *Firmicutes* to *Bacteroidetes* (F/B) ratio has been proposed as a microbial biomarker for hypertension [7]. We observed that F/B ratio was higher in the HF (9.19 ± 1.09) compared to control (3.19 ± 0.2; both *p* < 0.05) (Figure 3B). The increase of F/B ratio was prevented by prebiotic (3.37 ± 1.84%, *p* < 0.05 versus HF) as well as probiotic therapy (4.4 ± 1.33%, *p* < 0.05 versus HF). 

The main bacterial genera were shown in Figure 3C, including *Clostridium, Parabacteroides, Lactobacillus, Blautia, Akkermansia, Alkaliphilus, Sarcina, Ruminococcus, Flavobacterium, and Turicibacter*. We observed that HF diet caused a reduction of genus *Lactobacillus* abundance (6.36 ± 1.19 versus 10.56 ± 2.29; *p* < 0.05), which was prevented by probiotic therapy (14.98 ± 2.01; *p* < 0.05). The abundance of genus *Akkermansia* was lower in the HF group (3.2 ± 1.16%) compared to control (10.19 ± 2.31; *p* < 0.05) (Figure 3D). The decreased abundance of *Akkermansia* was restored by prebiotic as well as probiotic therapies (16.74 ± 3.61% and 9.2 ± 2.3%, both *p* < 0.05 versus HF).

**Figure 4:**
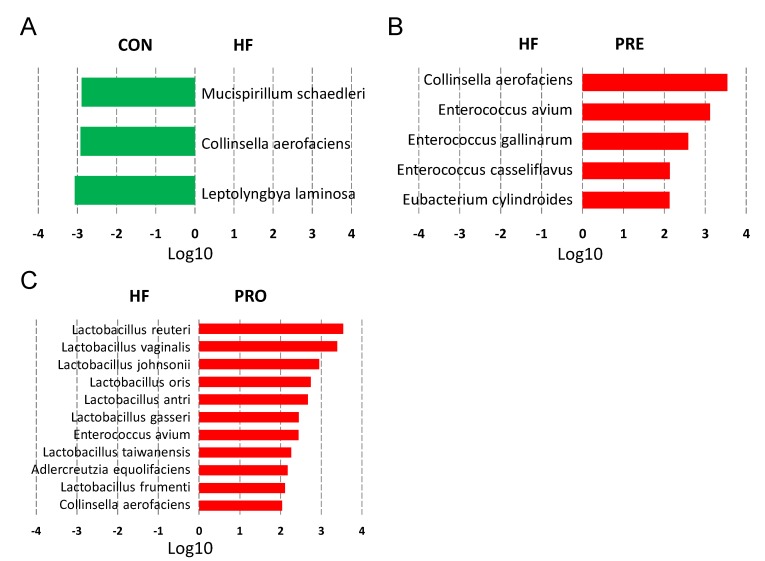
Effect of perinatal high-fat (HF) diet, prebiotic inulin (PRE), and probiotic *Lactobacillus casei* (PRO) on the gut microbiota at the species level at 3 weeks of age. Linear discriminant analysis (LDA), along with effect size measurements, was applied to identify enriched bacterial species. Most enriched and depleted species (LDA score (log10) > 2.0) in the (**A**) HF versus control (green), (**B**) PRE (red) versus HF, and (**C**) PRO (red) versus HF.

At 3 weeks of age, the main bacterial species modified by HF consumption were *Leptolyngbya laminose* (LDA score = −3.06), *Collinsella aerofaciens* (LDA score = −2.92), and *Mucispirillum schaedleri* (LDA score = −2.9) (Figure 4A). Prebiotic therapy increased abundance of species *Eubacterium cylindroides, Enterococcus casseliflavus, Enterococcus gallinarum, Enterococcus avium*, and *Collinsella aerofaciens* (Figure 4B). Importantly, HF-diet-induced reduction of several Lactobacillus species was prevented by probiotic therapy (Figure 4C). 

**Figure 5:**
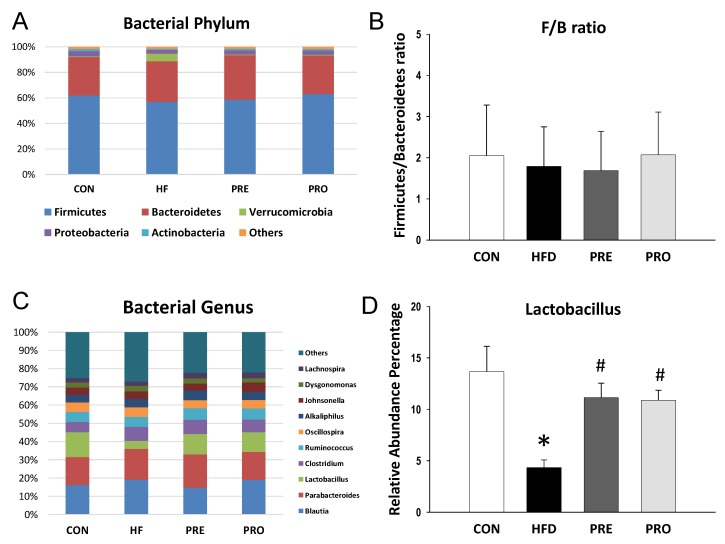
Effect of perinatal high-fat (HF) diet, prebiotic inulin (PRE), and probiotic *Lactobacillus casei* (PRO) on (**A**) relative abundances of the gut microbiota at the phylum level at 16 weeks of age; (**B**) the *Firmicutes* to *Bacteroidetes* ratio; (**C**) relative abundances of the gut microbiota at the genus level; and (**D**) the abundance of genus *Lactobacillus*. * *p* < 0.05 versus CON; # *p* < 0.05 versus HF.

As illustrated in Figure 5A, a similar trend of bacterial phyla was observed 3 and 16 weeks. Both alpha and beta diversity analysis showed similar trends at 16 weeks of age as observed at 3 weeks of age. Perinatal HF diet significantly reduced the abundance of the phylum *Proteobacteria* (2.9 ± 0.2% versus 4.1 ± 0.3%; *p* < 0.05), which prebiotic treatment prevented (3.5 ± 0.2%; *p* < 0.05). Additionally, the abundance of phylum *Actinobacteria* was lower in the HF (0.9 ± 0.1%), PRE (1.1 ± 0.1%), and PRO group (1.2 ± 0.1%) than that in the controls (1.7 ± 0.1%; all *p* < 0.05). Unlike at 3 weeks, the F/B ratio was not different among the four groups at 16 weeks of age (Figure 5B).

At the genus level, the top ten major genera were similar between 3- and 16-week gut microbiota (Figure 5C). Perinatal HF intake decreased the abundance of *Lactobacillus* (4.3 ± 0.8% versus 13.7 ± 2.5%, *p* < 0.05). The reduction of *Lactobacillus* abundance was restored by prebiotic (11.1 ± 1.4%, *p* < 0.05) or probiotic therapy (10.9 ± 1%, *p* < 0.05) (Figure 5D). Unlike at 3 weeks, the abundance of genus *Akkermansia* was not different among the four groups. At 16 weeks of age, the main bacterial species modified by HF consumption were *Leptolyngbya laminosa* (LDA score = −2.75), *Enterococcus avium* (LDA score = −2.32), and *Enterococcus casseliflavus* (LDA score = −2.01). Prebiotic therapy decreased abundance of species *Mucispirillum schaedleri* (LDA score = −2.42). 

## 4. Discussion

Our study describes, for the first time, prebiotic inulin or probiotic *Lactobacillus casei* protecting male offspring against hypertension programmed by perinatal high-fat diet, and puts special focus on the analysis of gut microbiota, microbiota-derived metabolites, and the RAS. Our major results can be summarized as follows: (1) perinatal HF diet induced elevation of BP in adult male offspring, which was prevented by either prebiotic or probiotic therapy; (2) maternal prebiotic therapy decreased fecal concentrations of propionate and acetate in offspring at 3 and 16 weeks of age, respectively; (3) prebiotic or probiotic therapy caused a reduction of plasma TMAO level and TMAO-to-TMA ratio; (4) HF diet increased renal mRNA expression of *Agt* and *Ace* and protein level of AT1R, which either prebiotic or probiotic therapy prevented; (5) perinatal HF diet increased the F/B ratio, and decreases of genera *Lactobacillus* and *Akkermansia* abundance in gut microbiota of 3-week-old offspring, all of which were restored by maternal microbiota-targeted therapy; and (6) probiotic therapy restored perinatal HF-diet-induced a reduction of several *Lactobacillus* species as well as genus *Lactobacillus* at 3 and 16 weeks of age, respectively. 

Our findings are in line with previous reports demonstrating that consumption of HF diet by pregnant dams causes programmed hypertension and kidney damage in their adult male offspring [28,29,30]. Although prebiotics and certain probiotic strains (e.g., *Lactobacillus*) have shown beneficial effect on hypertension-related disorders [31], to our knowledge, this is the first study to report maternal prebiotics/probiotics therapy prevented adult rat offspring from hypertension programmed by perinatal high-fat intake. In the current study, the BP-lowering effect of either probiotic or prebiotic therapy was starting from 12 weeks of age (i.e., 9 weeks after stopping probiotic or prebiotic therapy) and over time. Thus, the reduction of BP is primary through reprogramming effect rather than an acute effect. These findings support the notion that HF diet-induced early overnutrition results in hypertension in adulthood and 6-week administration of inulin or *Lactobacillus casei* during pregnancy and lactation could protect the development of hypertension later in life. Although prebiotic inulin or probiotic *Lactobacillus casei* showed similar BP-lowering effect in the present study, there might be a synergistic effect by combining prebiotic and probiotic therapies (i.e., synbiotics). It is also interesting to elucidate whether another modulation of gut microbiota targeted approach, fecal microbiota transplant, has beneficial effect on hypertension programmed by perinatal HF intake. 

This aspect of the research suggested that the anti-hypertensive effects of probiotic and prebiotic therapies link to alterations of the gut microbiota. Gut microbiota dysbiosis in early life may have a wide range of deleterious health consequences, including an increased risk of hypertension [32,33]. Although dietary fat has been suggested to be a causative factor with the gut microbiota dysbiosis in human and experimental studies [34], little attention has been paid to explore the impact of perinatal HF intake on the offspring gut microbiota [35,36]. Our results go beyond previous studies, showing that reshaping gut microbiota by maternal prebiotic or probiotic therapy could aid in overcoming hypertension programmed by HF diet-induced early overnutrition. At the phylum level, we observed that prebiotic or probiotic therapy prevented HF-induced hypertension is relevant to a reduced abundance of *Firmicutes* with a proportional increase in *Bacteroidetes* and *Verrucomicrobia*. The F/B ratio is associated with increased BP in several models of hypertension [7,37]. Our results here showed that perinatal HF diet increased the F/B ratio at 3 weeks of age, prior to the development of hypertension. Conversely, prebiotic or probiotic therapy decreased the F/B ratio and prevented adult male offspring against the rise in BP. However, the F/B ratio was comparable among the four groups at 16 weeks of age, a stage of established hypertension. Our findings suggest that the F/B ratio might serve as a microbial marker for predicting hypertension. 

According to our data, prebiotic or probiotic therapy protects adult offspring against elevated BP related to an increased abundance of phylum *Verrucomicrobia* and genera *Lactobacillus* and *Akkermansia*. This is consistent with what has been found in previous studies reporting *Akkermansia*, a genus in the phylum *Verrucomicrobia*, as a beneficial gut microbe [38,39]. Likewise, *Lactobacillus* has been reported to be one of the beneficial probiotic bacterial strains [40]. HF diet caused a reduction of genus *Lactobacillus*, whereas probiotic *Lactobacillus casei* therapy preserved the decreases of several *Lactobacillus* species caused by perinatal HF consumption. Additionally, results from a previous study demonstrated that *Collinsella aerofaciens* is increased in patients with coronary artery disease [41]. However, we observed that HF diet reduced *Collinsella aerofaciens*, which was reversed by prebiotic or probiotic therapy. Hence, it remains unclear whether alterations of gut microbiota act as a counterbalancing mechanism in response to perinatal HF diet or whether certain alterations of microbial populations interact directly with hypertension phenotype. Future research should further identify and confirm these microbial markers in other developmental programming models of hypertension. It has been reported that the composition of human gut microbiota changes with age [42]. We observed not only the alterations of gut microbiota compositions were different with age, but also some microbial makers (i.e., F/B ratio) appeared at 3 weeks while disappeared at 16 weeks of age. One possible reason is programming effects of perinatal high-fat diet and maternal probiotics/prebiotics therapy might be diminished as time went by. Another possibility is that all rats consumed the same post-weaning diet.

Emerging evidence supports that gut microbiota-derived metabolites such as SCFAs and TMAO are involved in BP regulation [12,18]. We observed that maternal inulin therapy decreases fecal propionate and acetate level at 3 and 16 weeks of age, respectively. Propionate and acetate have been reported to induce vasodilatation via mediating SCFA receptor [12]. Accordingly, decreased propionate or acetate level is presumed to induce rather than reduce BP. Whether SCFAs play a beneficial or harmful role in the development of hypertension programmed by perinatal HF diet remains to be clarified. 

Recent evidence reveals microbiota-derived metabolites TMAO and TMA related to cardiovascular disease [16,17], with not always consistent results [43]. Given that dietary factors are associated with plasma concentrations of TMA [16] and that TMAO level is controlled by its synthesis and metabolism, simultaneously measures of the TMAO-to-TMA ratio and the DMA-to-TMAO ratio were expected to reflect a cumulative state of TMAO. In the current study, perinatal HF diet caused the increases of TMA levels in adult offspring. Additionally, maternal probiotic or prebiotic therapy caused a lower TMAO-to-TMA ratio but a higher DMA-to-TMAO ratio than those in the HF group. These findings suggested the beneficial effects of prebiotic or probiotic treatment on HF-induced programmed hypertension are due to, at least in part, the prevention of TMAO accumulation. Consequently, more studies are required to simultaneous determinations of TMAO-related metabolites and ratios for their utility in predicting hypertension in other developmental models.

Furthermore, another possible beneficial effect of prebiotic and probiotic therapies is attributed to mediation of the RAS. In line with previous studies showing that high-fat intake activates the RAS [22,44], our results demonstrated that HF diet increased renal mRNA expression of *Agt* and *Ace*. Currently, there are two counterbalancing axes of the RAS: the classical ACE–Ang II–AT1R axis that promotes vasoconstriction and the non-classical ACE2–angiotensin (1–7)–MAS axis responsible for vasodilatation [13]. Our results cast a new light on the beneficial effects of prebiotic or probiotic therapy on HF-induced programmed hypertension is related to inhibition of the classical axis (i.e., *Ace* and AT1R expression) and activation of ACE2 in the non-classical axis. Since the presence of ACE-inhibitory peptides with anti-hypertensive effects have been reported during prebiotics and probiotic bacteria supplementation [45], the possibility of mediation of the RAS via prebiotic and probiotic therapies warrants further investigation.

This study has some limitations that have to be pointed out. First, we analyzed gut microbiota in offspring only at 3 and 16 weeks of age. The long-term effects of maternal prebiotic or probiotic therapy on offspring gut microbiota deserve further evaluation. Second, we did not analyze other organs responsible for BP regulation. The BP-lowering effect of probiotic or prebiotic might be related to other organs, such as the heart, brain, and vasculature. Another limitation is that we did not conduct the control group treated with prebiotic or prebiotic. The reason is due to that probiotics/prebiotics used in healthy people have only minor adverse effect, if any [40]. Moreover, we mainly focus on hypertension in this study. In addition to hypertension, maternal HF diet has been linked to a variety of metabolic syndrome-related phenotypes in adult offspring. Accordingly, whether maternal gut microbiota-targeted therapy may have beneficial effects on other HF-induced adverse metabolic diseases deserve further clarification. Probiotics and prebiotics have been shown to promote the release of the gut hormone glucagon-like peptide 1 (GLP1) [46,47], resulting in reduced food intake, improved glucose tolerance, and promoted weight loss in obese people [48,49]. Given that we did not record food consumption by mothers in the current study, effects of prebiotic or probiotic therapy on BP later in life could be due to reduced food intake of dams, which might be independent of gut microbiota. Last, the small number of animals included might not reveal a true effect.

## 5. Conclusions

The gut-kidney axis recently started gaining importance in the development of hypertension [50]. To the best of our knowledge, this is the first report of maternal prebiotic inulin or probiotic *Lactobacillus casei* therapy on HF diet-induced hypertension with a focus on gut microbiota and their metabolites; a result that casts a new light on applying microbiota-targeting therapies as a reprogramming strategy to prevent the developmental programming of hypertension. Future work is certainly required to develop and translate early-life microbiota-targeting therapies into clinical practice to prevent hypertension and alleviate hypertension-associated economic burden.

## Figures and Tables

**Table 1:** Quantitative real-time polymerase chain reaction primers sequences.

Gene	Forward	Reverse
*Ren*	5 aacattaccagggcaactttcact 3	5 acccccttcatggtgatctg 3
*Agt*	5 gcccaggtcgcgatgat 3	5 tgtacaagatgctgagtgaggcaa 3
*Ace*	5 caccggcaaggtctgctt 3	5 cttggcatagtttcgtgaggaa 3
*Rn18s*	5 gccgcggtaattccagctcca 3	5 cccgcccgctcccaagatc 3

*Ren* = Renin, *Agt* = Angiotensinogen, *Ace* = Angiotensin converting enzyme (ACE)-1, *Rn18s* = 18S ribosomal RNA.

**Table 2:** Morphological and biochemical values in different experimental groups.

Groups	CON	HF	PRE	PRO
BW (g)	576 ± 7	567 ± 8	595 ± 13	544 ± 15
Left kidney weight (g)	2.41 ± 0.05	2.21 ± 0.06	2.28 ± 0.05	2.22 ± 0.1
Left kidney weight/100 g BW	0.42 ± 0.01	0.39 ± 0.01	0.38 ± 0.01	0.41 ± 0.01
Systolic blood pressure (mm Hg)	142 ± 1	150 ± 1 *	141 ± 1 #	144 ± 1 #
Diastolic blood pressure (mm Hg)	65 ± 2	78 ± 3 *	66 ± 4 #	68 ± 2
Mean arterial pressure (mm Hg)	91 ± 1	102 ± 2 *	91 ± 3 #	93 ± 1 #
Creatinine (μM)	14.5 ± 0.9	16.2± 1.3 *	13.5 ± 1.6 #	14.6 ± 1

CON, regular chow; HF, perinatal high-fat diet; PRE, high-fat diet plus 5% inulin; PRO, high-fat diet plus *Lactobacillus casei*. BW, body weight; *n* = 8/group; * *p* < 0.05 versus CON; # *p* < 0.05 versus HF.

**Table 3:** Fecal concentrations of acetate, butyrate, and propionate at 3 and 16 weeks of age.

Group	CON	HF	PRE	PRO
3 weeks				
Acetate, mM/g feces	4.46 ± 0.47	5.12 ± 0.33	4.72 ± 0.37	5.72 ± 0.86
Propionate, mM/g feces	1.33 ± 0.38	0.49 ± 0.05 *	0.24 ± 0.04 * #	0.47 ± 0.08 * †
Butyrate, mM/g feces	0.45 ± 0.07	0.47 ± 0.04	0.4 ± 0.04	0.4 ± 0.07
16 weeks				
Acetate, mM/g feces	3.71 ± 0.14	3.66 ± 0.24	3.08 ± 0.21 *	3.89 ± 0.27 †
Propionate, mM/g feces	0.88 ± 0.05	0.76 ± 0.05	0.72 ± 0.06	0.78 ± 0.03
Butyrate, mM/g feces	1.67 ± 0.23	1.5 ± 0.31	1.14 ± 0.24	2.03 ± 0.31 †

*n* = 8/group; * *p* < 0.05 versus CON; # *p* < 0.05 versus HF; † *p* < 0.05 versus PRE.

**Table 4:** Plasma levels of trimethylamine N-oxide (TMAO), trimethylamine (TMA), and dimethylamine (DMA) at 16 weeks of age.

Group	CON	HF	PRE	PRO
TMAO, ng/mL	326 ± 18	328 ± 22	233 ± 15 *#	204 ± 7 * #
TMA, ng/mL	269 ± 15	363 ± 30 *	356 ± 31 *	395 ± 21 *
DMA, ng/mL	149 ± 4	127 ± 4 *	139 ± 6	115 ± 3 * # †
TMAO-to-TMA ratio	1.24 ± 0.11	0.93 ± 0.06 *	0.69 ± 0.08 *#	0.53 ± 0.03 * #
DMA-to-TMAO ratio	0.47 ± 0.03	0.4 ± 0.02	0.62 ± 0.06 *#	0.57 ± 0.03 * #

*n* = 7/group; * *p* < 0.05 versus CON; # *p* < 0.05 versus HF; † *p* < 0.05 versus PRE.
